# Social networks help to infer causality in the tumor microenvironment

**DOI:** 10.1186/s13104-016-1976-8

**Published:** 2016-03-15

**Authors:** Isaac Crespo, Marie-Agnès Doucey, Ioannis Xenarios

**Affiliations:** Vital-IT, SIB (Swiss Institute of Bioinformatics), University of Lausanne, Lausanne, Switzerland; Ludwig Center for Cancer Research, University of Lausanne, Epalinges, Switzerland

**Keywords:** Causality, Network inference, Social networks, Tumor microenvironment

## Abstract

**Background:**

Networks have become a popular way to conceptualize a system of interacting elements, such as electronic circuits, social communication, metabolism or gene regulation. Network inference, analysis, and modeling techniques have been developed in different areas of science and technology, such as computer science, mathematics, physics, and biology, with an active interdisciplinary exchange of concepts and approaches. However, some concepts seem to belong to a specific field without a clear transferability to other domains. At the same time, it is increasingly recognized that within some biological systems—such as the tumor microenvironment—where different types of resident and infiltrating cells interact to carry out their functions, the complexity of the system demands a theoretical framework, such as statistical inference, graph analysis and dynamical models, in order to asses and study the information derived from high-throughput experimental technologies.

**Results:**

In this article we propose to adopt and adapt the concepts of influence and investment from the world of social network analysis to biological problems, and in particular to apply this approach to infer causality in the tumor microenvironment. We showed that constructing a bidirectional network of influence between cell and cell communication molecules allowed us to determine the direction of inferred regulations at the expression level and correctly recapitulate cause-effect relationships described in literature.

**Conclusions:**

This work constitutes an example of a transfer of knowledge and concepts from the world of social network analysis to biomedical research, in particular to infer network causality in biological networks. This causality elucidation is essential to model the homeostatic response of biological systems to internal and external factors, such as environmental conditions, pathogens or treatments.

**Electronic supplementary material:**

The online version of this article (doi:10.1186/s13104-016-1976-8) contains supplementary material, which is available to authorized users.

## Background

Despite their differences in nature, social and biological networks are self-organising, emergent, and complex, and their respective analyses have common features: both focus on local and global patterns of connectivity, search for influential entities, and aim to model the network dynamics. Some concepts resulting from the study of social networks such as ‘popularity’, which refers to node centrality, can be directly transferred to the study of biological networks, where popular nodes are referred to as ‘hubs’, which tend to be essential [1]. However, there exist other concepts that seem to be more specific to social studies. Social network analysis has invested some efforts in describing network interactions at the so-called dyadic and triadic levels or the relationships between two (or, respectively, three) individuals, with the development of concepts such as social equality, balance, transitivity, and mutuality [2], which seem to be more specific to the realm of social studies. In particular, at the dyadic level, mutuality refers to the reciprocity between two individuals implicit in some types of social interactions, as for example the influence between individuals. Hangal et al. [3] proposed to model the influence of individual A over B in a given social graph as the fraction of B’s actions due to A, and the opposite for the influence of B over A. This influence is based on social interactions that imply a cost or investment for the people involved, and it is frequently asymmetric. For instance, keeping B posted by A requires time and effort that is considered an investment of A in B, and it reflects the fact that B has a certain influence on A, which can be very different than the influence of A on B.

**Fig. 1:**
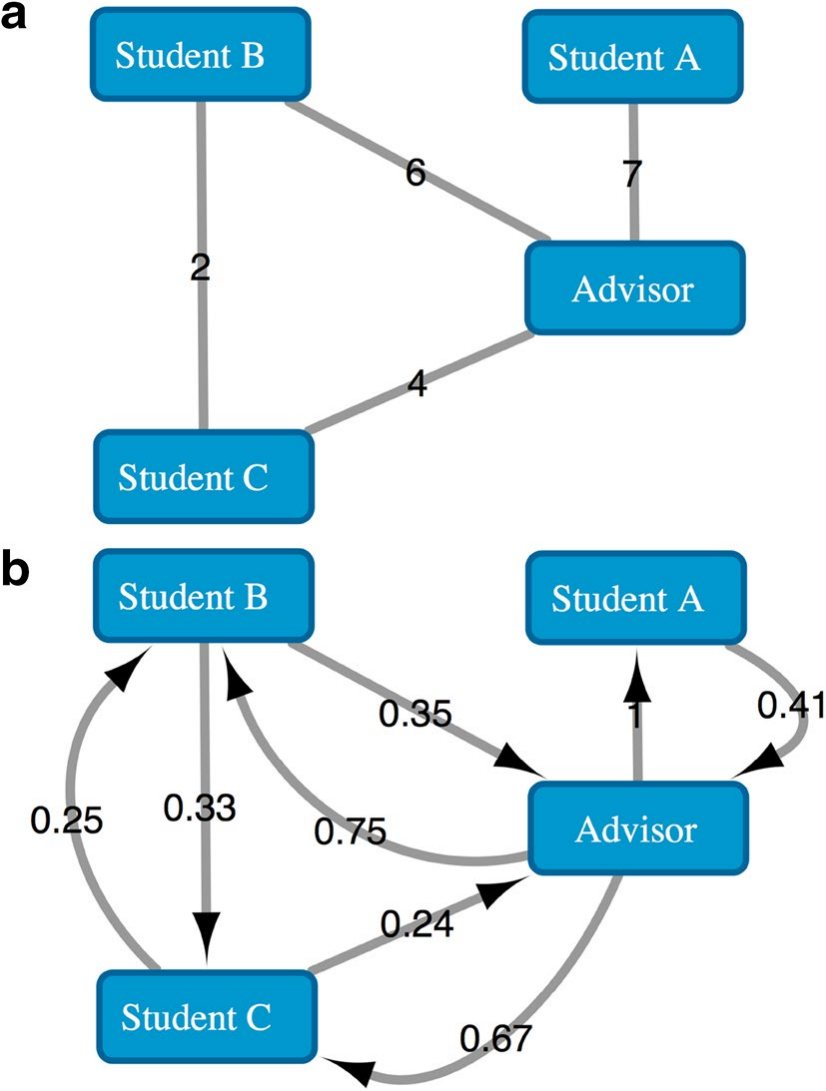
Modeling human interactions as an influence network. **a** Undirected and weighted social network where nodes represent three students and their advisor and edges represent shared publications. Edge weights represent the number of shared publications. **b** Derived bidirectional influence network. Weights represent the influence of the source on the target. The influence is calculated by dividing shared publication between source and target by other shared publications of the target with third parties

In some social graphs, the link between two individuals does not have directionality, as for example in an authorship graph, where authors are connected by shared publications (see Fig. 1). Hangal et al. [3] showed that this kind of graph can be derived in a bidirectional network of influence by assuming asymmetric mutuality pairwise, and demonstrated that such a derived network was more convenient for global social searches than methods based on the shortest path.

The construction of a directed network of influence based on an initially undirected graph is very attractive for biologists because in biomedical research there is an abundance of high-throughput experimental data that allows the construction of undirected correlation networks connecting different types of biological entities, such as genes or proteins, but the predictive power of these networks is limited due to their lack of causality or directionality. Based on the definition of influence proposed by Hangal et al. [3], Penrod et al. [4] developed a method for drug target discovery in the context of cancer therapy and showed that influential genes tend to be essential for the proliferation and survival of breast cancer cells, and that gene influence differs between untreated tumors and residual tumors that have adapted to a drug treatment. In order to calculate the investments between two genes, Penrod et al. [4] took the values of their partial correlation derived from expression data. It is worth noting here that despite Penrod et al. [4] having shown the utility of deriving the influence network from the co-expression information to identify genes essential for proliferation and survival of breast cancer cells, the underlying regulatory mechanisms involving these essential genes were not elucidated.

Using the concepts of investments and influencing mutuality from the social network analysis world, here we propose an approach to infer causality in co-expression networks derived from solid tumor expression data. In particular, we construct co-expression networks and infer cause-effect relationships between genes encoding cell–cell communication molecules as a model of the tumor microenvironment (TME) in breast, ovarian, and lung cancers. We show that constructing a bidirectional network of influence between cell–cell communication molecules allows us to determine the direction of cause-effect relationships underlying the correlation between genes (undirected in nature) and described in the literature.

Some methods have been proposed in the past to elucidate causality in biological networks inferred from experimental data, either from protein–protein interaction (PPI) information [5–7] or a combination of PPI and protein–DNA interactions [8] or perturbation experiments [9]. Specifically in the context of cancer, the partial least squares method was used to link the level of 19 proteins involved in apoptotic signaling in human colon adenocarcinoma cells to four quantitative measures of apoptosis [10]. The resulting directed network led to the prediction of cell death under specific perturbations.

In a previous study, we investigated the causality inference in the TME in breast cancer by constructing a dynamical model based on perturbation experiments [11]. The model was used to determine how to effectively revert the angiogenic activity of Tie-2 expressing monocytes. In general, strategies based on perturbation to infer causality are limited to small systems with a limited set of genes or proteins due to the combinatorial nature of the problem and its cost.

In the attempt to infer causality from correlation data, which is much more abundant than perturbation data, Gupta et al. [12] proposed a method that integrates network inference and network analysis approaches to construct co-expression networks and assign directionality to edges. The method was applied to time-series data to infer the topology of the gene regulatory network (GRN) of *B. subtilis.*

Instead of using the criterion proposed by Gupta et al. [12] to assign directionality to co-expression edges, in this work we adopted a similar criterion to assign directionality to the investments; the final directionality assignment for the co-expression edges, which represent the major regulatory effect, is based on the ratio of investments between two given nodes and investments that the investor has on other genes, as proposed by Hangal et al. [3] for social graphs. In doing so, and given that the ratio of investments is a topological property that does not rest on the dynamics of the system, costly time-series or perturbation experiments are not needed to elucidate the directionality of the interaction between two given genes. Consequently, our approach can take advantage of a wealth of expression data of comparative studies accumulated over decades of cancer research.

## Methods

### Principles of the approach

In biological systems, the circuitry of the underlying network can cause certain direct or indirect reciprocity on the regulatory effects performed by direct interactions; there exist regulatory feedback loops contributing to determine the dynamical behavior of living systems and maintaining their general homeostasis. In other words, in order to guarantee a certain level of homeostasis, the constitutive elements of biological networks should be capable of mutually affecting each other directly or indirectly.

The approach presented here for causality inference is based on the following assumption: given two genes A and B in a regulatory network, if there exists a direct effect from A to B, most of the indirect effects between these two nodes will have the opposite direction (from B to A). In this context, indirect/direct effect refers to an effect with/without intermediates belonging to the considered network. The rationale behind this assumption is the idea that homeostatic control requires reciprocity and given that the direct interaction covers one direction, the indirect interactions should have the opposite direction. Of course, such an assumption neglects feed-forward loops, which are well-known regulatory mechanisms [13–15].

In order to infer network causality, the above-mentioned assumption was combined with network inference based in partial correlation [16] and the adopted concepts of influence and investments proposed by Hangal et al. [3].

### Algorithm description for directionality assignment

**Fig. 2:**
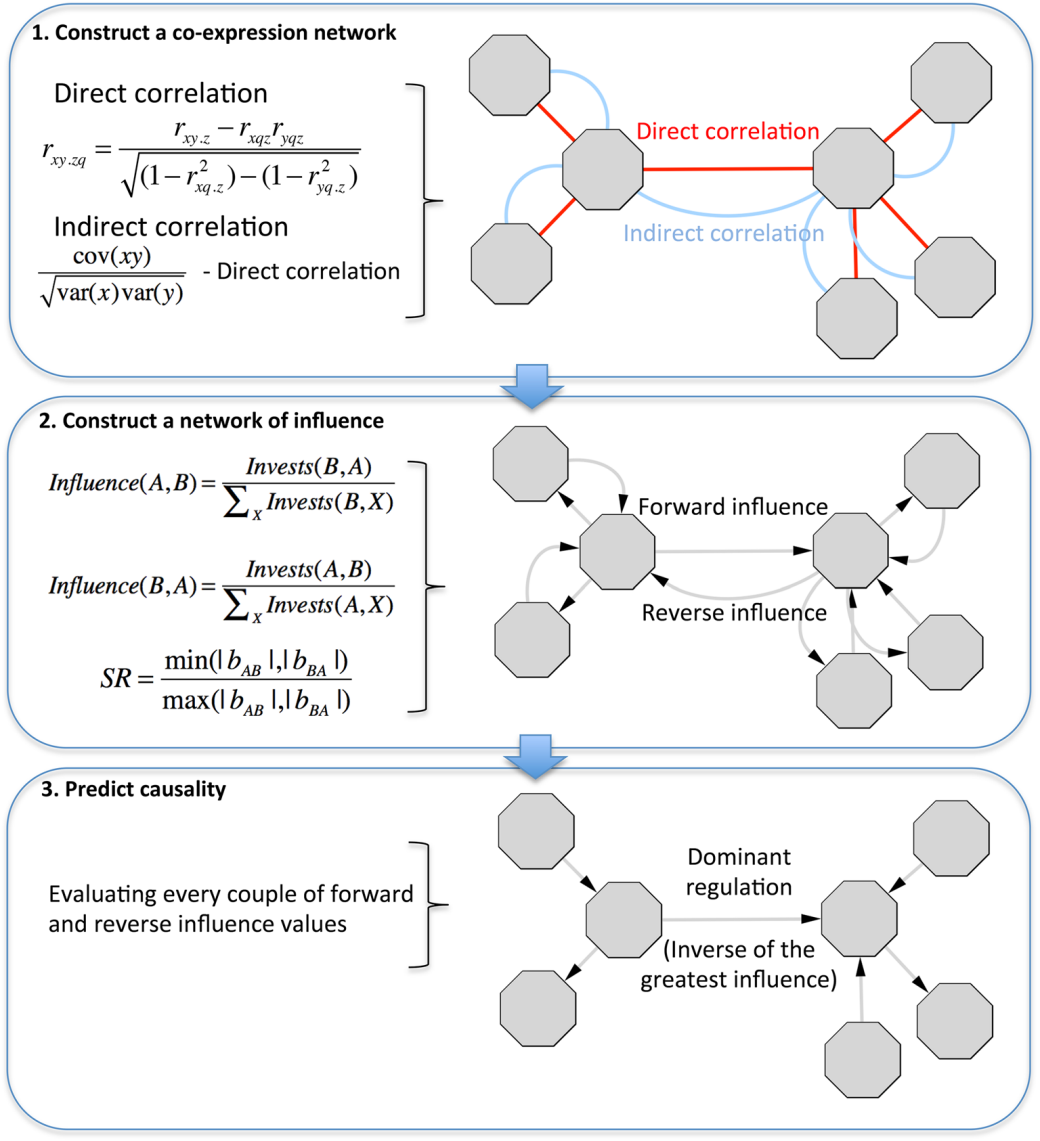
Algorithm description in three steps. In the first step, a co-expression network is constructed based on the calculation of direct and indirect correlation. Direct correlation refers to the second-order partial correlation, whereas indirect correlation is calculated as the difference between zeroth-order partial correlation and the direct correlation. In the second step, a bidirectional network of influence is constructed. Forward and reverse influences are derived from the calculation of the investments for each couple of genes. The directionality assignment of the investments is based on the slope ratio (SR) criterion. In the third step, causality is inferred by evaluating every couple of forward and reverse influences. The directionality of the predictions is the opposite of the highest influence value and can be interpreted as the directionality of the main flux of regulatory effects

The algorithm can be described in three steps (see Fig. 2):

#### Construct a co-expression network

To this end, we used the software package Ometer (http://www.comp-sys-bio.org/Ometer.html). Between any two given genes we calculated the partial correlation based on the Pearson coefficient. The partial correlation has been previously proposed to discover associations in genomic data [16]. It quantifies the correlation between two variables (in our case, gene expression) when conditioning on one or several other variables. The order of the partial correlation is determined by the number of variables it is conditioned on; within this work we used up to second-order partial correlation. Equations (1–3) allow the calculation of partial correlations of orders 0–2.

1 $${\text{Zeroth-}}{\text{order}}:r_{{xy}} = \frac{{\text{cov} \left( {xy} \right)}}{{\sqrt {\text{var} \left( x \right)\text{var} \left( y \right)} }}$$

2 $${\text{First-order}}: \, rxy \cdot z = \frac{{r_{xy} { - }r_{xz} r_{yz} }}{{\sqrt {\left( {1{ - }r_{xz}^{2} } \right){ - }\left( {1{ - }r_{yz}^{2} } \right)} }}$$

3 $${\text{Second-order}}:r_{xy \cdot zq} = \frac{{r_{xy.z} { - }r_{xqz} r_{yqz} }}{{\sqrt {\left( {1{ - }r_{xq \cdot z}^{2} } \right){ - }\left( {1{ - }r_{yq.z}^{2} } \right)} }}$$

The algorithm further considered only interactions with a p value below 0.05. Results using different thresholds (p values of 0.1 and 0.01) are included in the “Results” section.

At this point, the algorithm will make an assumption: given two genes A and B, if there exists a direct effect from A to B, most of the indirect effects between these two nodes will have the opposite direction (from B to A). The rationale behind this assumption is described above in principles of the approach. Following this assumption, the algorithm establishes that partial correlation gives us the strength of the effect in one direction and the difference between correlation and partial correlation (correlation–partial correlation) gives us the strength of the effect in the opposite direction.

**Fig. 3:**
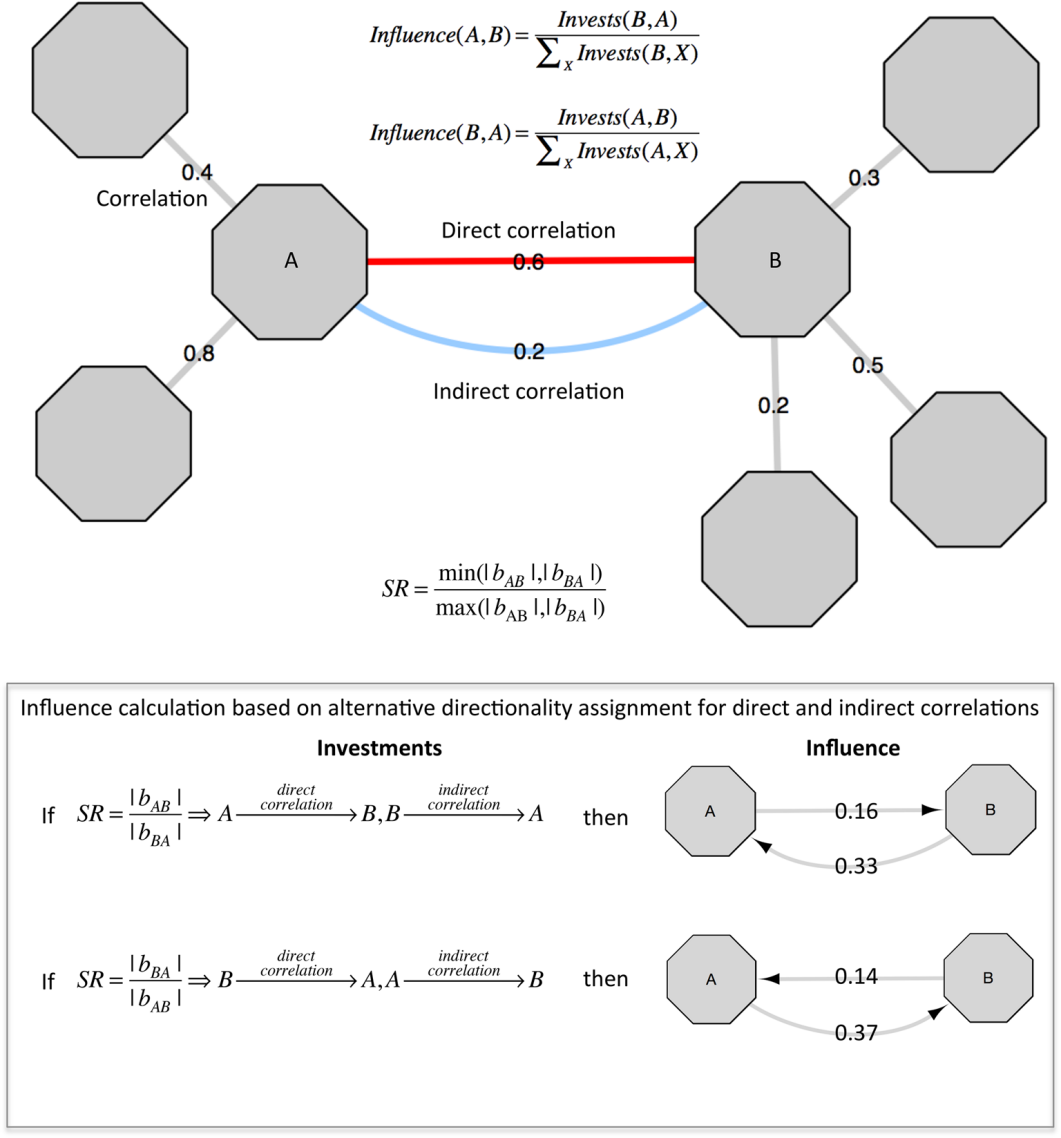
Influence calculation. Forward and reverse influence calculation between two genes A and B. Links in *grey* represent investments of A and B on other genes. The assignment of directionality to direct and indirect correlation is based on the slope ratio (SR) criterion

From now on, we will refer to the partial correlation as direct correlation and to the difference between correlation and partial correlation as indirect correlation, which has opposite direction than direct correlation. There are two possible directionality assignments for direct and indirect correlation between two given nodes A and B (see Fig. 3) and both will be further evaluated for each couple of nodes with statistically significant correlation.

#### Construct a network of influence

We adopted the concepts of investments and influence proposed by Hangal et al. [3] to construct a weighted bidirectional network of influence. Investments will be the numerical value of the direct and indirect correlation, and the influence will be calculated dividing the investments between A and B by the total investments of the investors, i.e., B for forward influence (A → B) and A for reverse influence (A ← B). Given that we do not know if the direct (conversely, indirect) correlation is associated with either forward (A → B) or reverse (A ← B) influence, we also do not know whether we should divide the direct and indirect correlation by the investments of A or B. Moreover, in order to calculate the investments of the investor on other genes we also need to assign either values of direct or indirect correlation to the outgoing interactions of the investor. At this point, the algorithm will assign the value of direct and indirect correlations based on the so-called slope ratio metric (SR) following the strategy proposed by Gupta et al. [12]. The SR is defined as

$$SR = \frac{{\hbox{min} \left( {\left| {\left. {b_{yx} } \right|} \right.,\left| {\left. {b_{xy} } \right|} \right.} \right)}}{{\hbox{max} \left( {\left| {\left. {b_{yx} } \right|} \right.,\left| {\left. {b_{xy} } \right|} \right.} \right)}}$$

*b*_*YX*_ and *b*_*XY*_ represent the regression slopes of a pair of variables (gene expression values).

Gupta et al. proposed the following rules in order to assign directionality to correlation edges only for those edges that have *SR* → 0

$${\text{If}}\,SR = \frac{{\left| {\left. {b_{YX} } \right|} \right.}}{{\left| {\left. {b_{XY} } \right|} \right.}} \Rightarrow Y \to X$$

$$\begin{aligned} {\text{If}}\,SR = \frac{{\left| {\left. {b_{XY} } \right|} \right.}}{{\left| {\left. {b_{YX} } \right|} \right.}} \Rightarrow X \to Y \hfill \\ \hfill \\ \end{aligned}.$$

Our algorithm uses the same set of rules to assign the values of direct and indirect correlation to incoming and outgoing edges

$${\text{If}}\,SR = \frac{{\left| {\left. {b_{YX} } \right|} \right.}}{{\left| {\left. {b_{XY} } \right|} \right.}} \Rightarrow Y\underrightarrow {direct - correlation}\,X,X\underrightarrow {indirect - correlation}\,Y$$

$${\text{If}}\,SR = \frac{{\left| {\left. {b_{XY} } \right|} \right.}}{{\left| {\left. {b_{YX} } \right|} \right.}} \Rightarrow X\underrightarrow {direct - correlation}\,Y,Y\underrightarrow {indirect - correlation}\,X$$

The intuitive idea behind the decision of the algorithm at this point is that the direct correlation (partial correlation) is always assigned to the link from the gene with a smaller variance to the gene with a bigger variance, given that

$$b_{YX} = \frac{{\sum {_{i = 1}^{N} \left( {X_{i} { - }\bar{X}} \right)} \cdot \left( {Y{ - }\bar{Y}} \right)}}{{\sum {_{i = 1}^{N} \left( {X_{i} { - }\bar{X}} \right)^{2} } }}$$

$$b_{XY} = \frac{{\sum {_{i = 1}^{N} \left( {X_{i} { - }\bar{X}} \right)} \cdot \left( {Y{ - }\bar{Y}} \right)}}{{\sum {_{i = 1}^{N} \left( {Y_{i} { - }\bar{Y}} \right)^{2} } }}$$

It is worth noting here that this assignment is not related to the weight of the link; sometimes the direct correlation is stronger than the indirect correlation and sometimes it is the opposite.

Once the algorithm has calculated the outgoing investments for every gene in the network, the influence bidirectional network is easily derived applying the following formula for each couple of genes A and B

$$Influence\left( {A,B} \right) = \frac{{Invests\left( {B,A} \right)}}{{\sum {_{X} Invests\left( {B,X} \right)} }}$$

$$Influence\left( {B,A} \right) = \frac{{Invests\left( {A,B} \right)}}{{\sum {_{X} Invests\left( {A,X} \right)} }}$$

X refers to all the genes targeted by the investor. It is worth noting here that when calculating the influence, positive and negative investments (consequence of positive and negative correlations) are divided correspondingly by positive and negative investments of the investor.

#### Predict causality

Once the forward and reverse influence between any couple of nodes in the influence network has been calculated, the algorithm compares both values and selects the biggest one as the main flux of influence. The directionality predictions for the original co-expression network will have the opposite direction than the main flux of influence. These predictions can be interpreted, according to the adopted definitions, as the direction of the main flux of regulatory effects (dedicated investments). Empirically, we noticed that percentages of correct directionality assignments were improved when discarding the lowest influence values. Accordingly, we systematically discarded the lowest 25 % of values for the three examples presented in this work.

### Example datasets

In order to construct the correlation networks we use microarray expression data from public repositories. Datasets for breast and ovarian cancer (1809 and 1394 patients respectively) were downloaded from the KM plotter website (www.kmplot.com), whereas the dataset for lung cancer (688 patients) was constructed using the following references from GEO database: GSE14814, GSE19188, GSE31210 and GSE 37745. The raw CEL files were MAS5 normalized in the R statistical environment (www.r-project.org) using the Affy Bioconductor Library [17]. The three datasets also have a second scaling normalization to set the average expression on each chip to 1000 to avoid batch effects [18]. The datasets were obtained using either HG-U133A (GPL96) or HG-U133 Plus 2.0 (GPL570). These platforms include 283 probes corresponding to 192 genes annotated as cytokines, cell–cell communication molecules or growth factors according to GO database (http://geneontology.org). Consequently, the expression values of these 283 probes were summarized into 192 numerical values by calculating the mean of probes referring to the same gene.

### Evaluation of predictions

Predictions were evaluated using directed cause-effect relationships contained in the ResNet database from Ariadne Genomics (http://www.ariadnegenomics.com/). We selected only the interactions included in the ResNet mammalian database in the category of ‘Expression’. Interactions in the ‘Expression’ category indicate that the expression of regulatory gene/protein affects their targets, by (both directly and indirectly) regulating its gene expression or protein stability. The ResNet database includes biological relationships and associations which have been extracted from the biomedical literature using Ariadne’s MedScan technology [19, 20]. MedScan processes sentences from PubMed abstracts and produces a set of regularized logical structures representing the meaning of each sentence. ResNet was queried looking for interactions between 192 genes annotated as cell–cell communication molecules or growth factors. It is worth noting here that no filter was applied based on the biological context. That means that the interactions included have been described in a variety of cell types, tissues and other experimental conditions, and thus are not restricted to observations in a tumor context. We obtained 1774 interactions directed and signed (either activation or inhibition). The complete list of interactions with their respective references is included in the Additional files 1, 2. For the evaluation of directionality assignment, we only considered predictions involving couples of genes previously described to be interacting. The number of such interactions reported in literature is different for each example (see Table 1 in "Results" section). When the predicted directionality matched directionality reported in the literature, it counted as ‘correct’; if directionality did not match the interaction it counted as ‘incorrect’. The percentage of correct directionality assignment refers to the ratio between ‘correct’ and ‘total’ (‘correct’ plus ‘incorrect’) predictions multiplied by 100.

**Table 1:** Percentages of correctly predicted cause-effect relationships in breast (BC), ovarian (OC) and lung cancer (LC)

	Predicted interactions	Evaluated predictions	Correct predictions	Correct predictions (%)	p value
BC 0.01	1390	121	77	*63.63*	0.02
BC 0.05	1999	179	106	59.21	0.15
BC 0.1	2416	212	126	59.43	0.10
OC 0.01	891	106	66	*62.26*	0.04
OC 0.05	1223	139	81	58.27	0.21
OC 0.1	1465	160	97	60.62	0.04
LC 0.01	545	69	48	*69.56*	0.00
LC 0.05	909	93	60	64.51	0.05
LC 0.1	1207	115	67	58.26	0.24

The application of the method using p values of 0.01, 0.05 and 0.1 to construct the co-expression network resulted in different number and percentages of correct predictions. The most stringent p value (0.01) obtained the best percentages in BC, OC and LC datasets (in italics)

However, there is an issue in the procedure we used to evaluate the directionality assignment that has to be taken into account. Couples of genes mutually regulated according to literature will be correctly predicted whatever directionality is assigned (both directions are correct). Due to that, the random directionality assignment may be right more than the expected 50 % of the time.

**Fig. 4:**
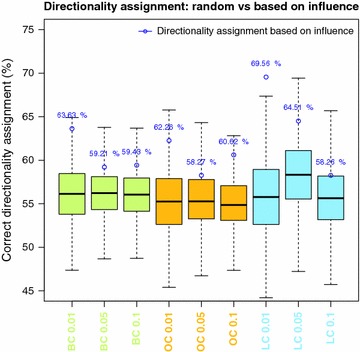
Comparison between influence-based and random directionality assignment in breast, ovarian and lung cancer. The directionality assignment based on influence (*blue dots*) performed better than random for the three biological examples, namely breast, ovarian and lung cancer (*green*, *orange* and *blue* *boxplot* respectively). The best results in the three cases were obtained using a p value of 0.01 as the threshold for the co-expression network construction (labeled as BC 0.01, OC 0.01 and LC 0.01)

In order to obtain a fair comparison of our method against random directionality assignment (see Fig. 4), we generated a population of 10,000 assignments with the same probability of 0.5 for one direction and the opposite for each couple of genes with statistically significant partial correlation between them and for the three case examples. The random directionality assignment performed slightly better than 50 % for the three case examples and differently depending on the subset of gene couples used for the evaluation (see Fig. 4), which ultimately depend on the p value of their partial correlation and the specific example. Consequently, the scores (% of correct assignments) of these populations of alternative random assignments can be justifiably compared against the ones obtained by our algorithm.

## Results

We applied the proposed methodology to the TME because of the paramount importance of causality to develop novel combined cancer therapies. Breast, ovarian and lung cancer were selected because of the abundance of publicly available datasets with both expression and clinical data.

Some aspects of tumor biology, such as pro-angiogenic and immune suppressive states, rely on cell–cell communication events; internal cellular processes are significantly influenced by the interplay between different cells types carried out through cell–cell communication molecules, which become potential targets of novel therapies. However, the complexity of the TME demands theoretical frameworks, such as statistical inference, graph analysis and dynamical models, in order to assess and study the information derived from high-throughput experimental technologies. A predictive model of the TME should capture interdependencies between tumor microenvironment components and predict their response to single and combined perturbations, and will serve to identify the most efficient treatment combinations that induce desired cell properties, such as anti-angiogenic and immune-competent states, in the TME. Such a model requires directionality or causality when describing interdependencies between TME components. Statistical analysis of concentrations of cell–cell communication molecules in tumor samples allows the construction of a correlation network at the level of gene products or gene expression. Unfortunately, correlation networks are undirected; a statistically significant correlation between genes ‘A’ and ‘B’ does not indicate if ‘A’ levels are causing ‘B’ levels or vice versa.

The methodology proposed in this work assigns directionality to co-expression edges based on the ratio of investments between two given nodes and investments that the investor has on other genes (see “Methods” section). Calculating the ratio of investments of the network of influence does not require costly time series and perturbation experiments.

In summary, we have applied an analysis to infer causality in the TME at the gene expression level across three different cancer types. Both co-expression and influence networks are included in the Additional file 1: Tables S1.

### Causality inference at the level of gene expression in breast, ovarian and lung cancer

After applying the methodology described in the “Methods” section to three different datasets derived from breast, ovarian and lung cancer patients, we observed that the directionality assignment based on influence performed better than a random assignment in the three cases (see Fig. 4). Different p values (0.01, 0.05 and 0.1) were used as the threshold for the inference of the initial co-expression network in order to evaluate the effect of this parameter on the directionality assignment. In the three cases, the best accuracy corresponded to the most stringent p value (0.01), resulting in correct predictions of 63.6, 62.2, and 69.5 % for breast, ovarian and lung cancer datasets respectively.

These percentages were estimated using information from literature about predicted interactions. Relaxing the stringency on the p value to construct the co-expression network allowed a larger amount of correctly predicted directionality assignment to be obtained, but with lower accuracy (see Table 1). Indeed, we observed that using a p value of 0.01 for the co-expression network as threshold we always obtained a statistically significant difference between the score of the influence-based network and randomly generated networks, with z-scores of 2.03, 1.77 and 2.81 for breast, ovarian and lung cancer respectively and p values <0.05, whereas for less stringent p values the difference was not always statistically supported (see Table 1).

## Discussion

The most prevalent use of network inference in biomedicine takes advantage of information such as gene expression or protein–protein binding data to predict the network topology as a set of correlations or physical interactions between its constitutive elements. Unfortunately, the resulting networks lack directionality, i.e., causality, hindering the elucidation of regulatory mechanisms and the flow of information through signaling pathways. This causality elucidation is essential to model the homeostatic response of the TME to internal and external factors and to predict the network response to perturbations such as targeted therapy. In order to elucidate this causality, one may consider dedicated perturbation experiments or time-series data, which are costly and not as abundant as comparative studies between a reduced set of conditions.

In this work we addressed the following question: To what extent can the description of a biological system (TME) in terms of influence and investments derived from social network analysis be useful to infer causality from comparative studies?

To answer this question, we developed a systems approach where network causality in the TME is inferred based not only on local properties of the system (such as the co-expression of two genes) but also on the global network topology analysis. We showed that the application of the strategy proposed here to breast, ovarian and lung cancer datasets allowed the prediction of causality from derived co-expression networks by constructing a network of influence and analyzing its topological properties. The resulting directionality assignments were evaluated using information from literature and compared across the three cancer types. Results showed that 63.63, 62.26 and 69.56 % of directionality predictions for breast, ovarian and lung cancer respectively were correct according to cause-effect relationships described in literature. These results indicated that costly perturbation experiments and time series data could be avoided while still providing a good approximation of the network causality.

The methodology described in this work does not address the other significant challenge in network inference: the appearance of false positives or correlation inferred due to the spurious co-occurrence of biological events. The transformation of the correlation network into an influence network will assign weights and directionality to both true and false interactions. Conversely, if there is no link between two given nodes in the correlation network, no new interactions will be inferred; false negatives or real interactions not included in the correlation network will not be further considered. In other words, the methodology presented in this work assumes the correlation networks are essentially correct and complete. Consequently, the causality inference technique proposed here will benefit from the further development of advanced methods for correlation network inference with high sensitivity and specificity.

As it is mentioned in “Methods” section, the adopted assumption to identify the direction of the dominant regulation between two genes somehow neglects the importance of feed-forward loops, which are well-known regulatory mechanisms [13–15]. However, the potential overestimation of the strength of the investments assigned with the direct correlation does not prevent the correct directionality assignment for the majority of gene pairs.

It is worth noting here that usually network inference techniques attempt to remove indirect interactions, or interactions through intermediaries, by using partial correlations [16, 21–23], conditional mutual information [24, 25] or data processing inequality [26, 27]. In this work we showed that considering not only direct but also indirect correlations (between genes that also have direct correlations) helped to predict directionality in three different case studies.

## Conclusions

This work constitutes an example of interdisciplinary transfer of concepts between the fields of social and biological network analysis that allows the development of a novel systems approach to infer network causality in the TME.

The main strength of this method is that it relies on experimental information from comparative studies, rather than costly dedicated perturbation experiments and time series data, usually required to infer cause-effect relationships.

The application of our method can help the experimental design, elucidation of regulatory mechanisms and identification of novel targets in cancer therapy and beyond.

## Additional files

10.1186/s13104-016-1976-8 xlsx. Networks.
10.1186/s13104-016-1976-8 Results influence-based network vs. random networks.

## Abbreviations

GRN: gene regulatory network
TME: tumor microenvironment
